# Neurofibromatosis type-1 is a prognostic indicator in human gastric carcinoma

**DOI:** 10.18632/oncotarget.19876

**Published:** 2017-08-03

**Authors:** Debao Liu, Yueying Zhang, Yan Li, Kaixi Fan

**Affiliations:** ^1^ Department of Oncology, Affiliated Hospital of Shandong Academy of Medical Sciences, Jinan, Shandong, China; ^2^ School of Medicine and Life Sciences, University of Jinan-Shandong Academy of Medical Sciences, Jinan, Shandong, China; ^3^ Department of Pathology and Pathophysiology, Institute of Basic Medicine, Shandong Academy of Medical Sciences, Jinan, Shandong, China; ^4^ Department of Central Laboratory, Affiliated Hospital of Shandong Academy of Medical Sciences, Jinan, Shandong, China

**Keywords:** gastric cancer, neurofibromatosis type I, TNM stage, immunohistochemistry, prognosis

## Abstract

We investigated whether the *Neurofibromatosis type-1*(*NF1*) gene was of prognostic relevance to gastric cancer (GC) patients. Immunohistochemical staining of 160 matched GC tumor and adjacent normal tissue samples showed that 58.1% (93/160) of GC samples were NF1-positive as compared to 94.4% (151/160) of normal tissue samples (χ^2^=58.05, *P* <0.001). qRT-PCR analysis revealed that NF1 mRNA expression is lower in GC tissues than normal tissues (χ^2^=34.23, *P* <0.001). Moreover, NF1 protein and mRNA levels were associated with T stage (*P* <0.05) and TNM (*P* <0.001). No association was observed with other clinicopathological parameters, including gender, age, tumor size, lymph-node metastasis, cancer differentiation and distant metastasis (all *P* >0.05). Kaplan-Meier analysis revealed that negative or low NF1 were associated with poor overall survival (OS) in gastric cancer patients (*P*<0.001). Further univariate and multivariate cox regression analysis also showed that NF1 expression was an independent risk factor of survival of GC patients. These data show that NF1 has prognostic relevance to clinical outcomes in gastric cancer patients.

## INTRODUCTION

Gastric cancer (GC) is the third leading cause of cancer-related deaths and the fourth most common malignant tumors [1]. Most GC patients are diagnosed at an advanced stage with lymph nodes metastasis [2]. Most patients experience metastasis and recurrence after undergoing standard treatment that includes adjuvant chemoradiotherapy and surgery. Hence, the 5-year survival rate of GC is very poor [3, 4]. Therefore, new prognostic tumor markers and therapeutic targets are necessary to improve the clinical outcomes for GC patients.

Neurofibromatosis type-1(NF1) or von Recklinghausen disease is a common autosomal dominant condition affecting the nervous system with an estimated incidence of about 1 in 3,000-4,000 individuals worldwide [5, 6]. It is also a multi-system disease associated with many cancers [7, 8]. The *NF1* gene is a classic tumor suppressor gene located on chromosome 17q11.2 and its product neurofibromin is an important negative regulator of Ras signaling pathway [9, 10]. Mutations in *NF1* gene result in NF1 disease. Many studies have demonstrated that *NF1* plays an important role in many cancers such as brain tumors [11], breast cancer [12], sporadic colon cancer [13], lung cancer [14], pheochromocytomas [15] and ovarian tumors [16]. Moreover, Vizcaino *et al.* demonstrated that glioma patients with low NF1 expression were associated with poor overall survival and disease-specific survival [17]. In this study, we investigated the relationship between *NF1* expression and clinicopathological characteristics of GC patients. Further, we analyzed the prognostic relevance of NF1 for GC patients.

## RESULTS

### NF1 protein expression in human gastric cancer tissues

**Figure 1:**
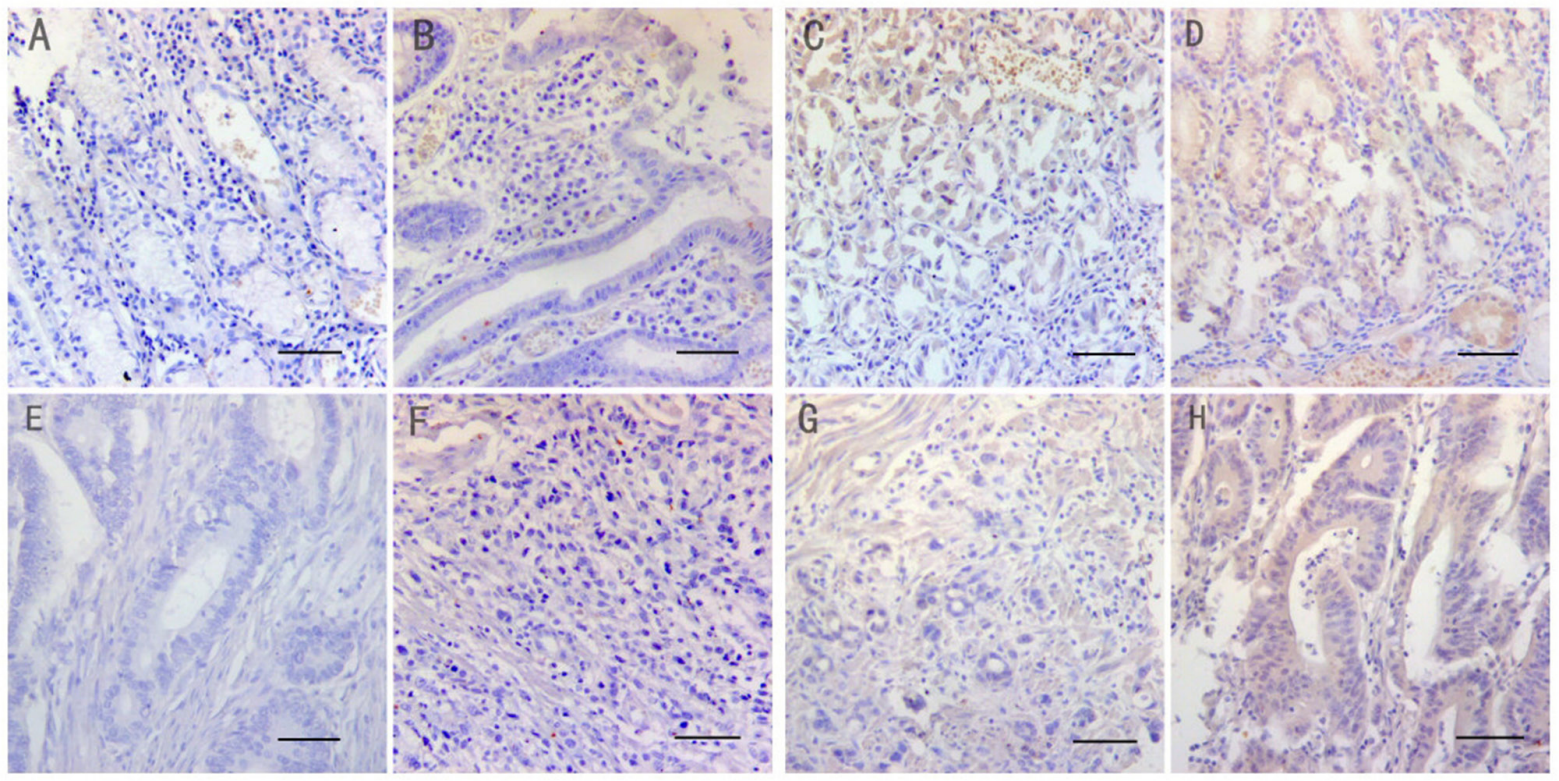
Immunohistochemical staining of NF1 in matched normal and gastric cancer tissues (Magnification: x200. Bar 200μm.) The micrographs show negative **(A&E)**, weak **(B&F)**, moderate **(C&G)** and strong **(D&H)** staining of NF1 in normal **(A-D)** and cancer tissues **(E-H)**, respectively.

First, we analyzed NF1 protein expression in 160 GC and paired non-cancerous tissues. We observed that the NF1 protein was mainly located in the cytoplasm. Its expression was higher in the normal gastric tissues (94.4%) compared to GC tissues (58.1%) (Table 1; χ2=58.05, *P* <0.001). Moreover, higher number of GC samples (59/93) demonstrated lower NF1 expression compared to normal gastric tissues (25/151; χ2=56.04, *P*<0.001). However, we did not find any difference in NF1 expression among the superior differentiation (well-differentiated tumors), moderate differentiation (moderately differentiated tumors) and the inferior differentiation (poorly differentiated and mucinous adenocarcinoma) groups (χ^2^=3.429, *P*=0.489). Representative immunohistochemical images of NF1 protein expression in normal and cancer tissues are presented in Figure 1.

**Table 1:** NF1 protein expression in normal and gastric cancer tissues based on immunohistochemistry

Tissue type	Total (n)	NF1 positive	% NF1 positives	*P value*
Normal	160	151	94.4	<0.001
Cancer	160	93	58.1	

### Relation between NF1 protein expression and clinicopathological features

We further divided the 160 cancer tissues into high, low and negative NF1 protein expression groups and assessed the correlation between NF1 expression and clinicopathological characteristics. Our data demonstrated that NF1 expression was associated with T stage (χ^2^=15.36, *P*=0.018) and TNM stages (χ^2^=27.39, *P*<0.001; Table 2). However, as shown in Table 2, NF1 was not associated with gender (*P*=0.693), age (*P*=0.971), tumor size (*P*=0.959), lymph-node metastasis (*P*=0.667), cancer differentiation (*P*=0.489) and distant metastasis (p=0.966).

**Table 2:** Correlation between NF1 protein expression and clinicopathological characteristics of GC patients

Characteristics	n (%)	NF1 protein expression (case)			*P value*
		High (n=34)	Low (n=59)	Negative (n=67)	
Age (years)					
<60	64(40)	13	24	27	0.971
≥60	96(60)	21	35	40	
Gender					
Male	104(65)	23	40	41	0.693
Female	56(35)	11	19	26	
Tumor size (cm)					
<5	91(57)	19	33	39	0.959
≥5	69(43)	15	26	28	
Distant metastasis					
Yes	16(10)	3	6	7	0.966
No	144(90)	31	53	60	
T stage					
T1	16(10)	9	5	2	0.018
T2	21(13)	5	7	9	
T3	92(58)	16	36	40	
T4	31(19)	4	11	16	
N stage					0.667
N0	48(30)	10	19	19	
N1	63(40)	15	25	23	
N2	31(19)	7	10	14	
N3	18(11)	2	5	11	
Differentiation					
Superior	6(4)	3	1	2	0.489
Moderate	45(28)	10	17	18	
Inferior	109(68)	21	41	47	
TNM stage					
I	25(16)	15	4	6	<0.001
II	67(42)	9	26	32	
III	52(32)	7	23	22	
IV	16(10)	3	6	7	

### Relationship between NF1 protein expression and the prognosis of these gastric cancer patients

**Figure 2:**
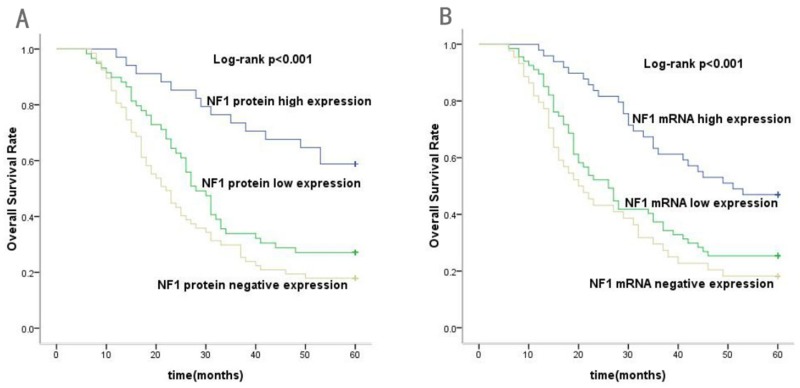
Kaplan–Meier analysis for overall survival (OS) of gastric cancer patients according to the NF1 protein and mRNA expression **(A)** Association of high, low or negative NF1 protein expression with OS (P<0.001). **(B)** Association of high, low or negative NF1 mRNA expression with OS (P<0.001).

At the end of the five-year follow-up (median,45months), 48 out of 160 patients were alive. Among them, 36 patients that showed NF1 protein expression had better prognosis compared to 12 patients with negative NF1 protein expression (χ2=8.023, *P*=0.005). In addition, among the 36 patients showing positive NF1 expression, 20 had high NF1 protein levels compared to 16 with low NF1 protein (χ2=9.139, *P*=0.003). The Kaplan-Meyer survival analysis results demonstrated that GC patients with high NF1 protein expression had a longer five-year overall survival than GC patients with low or negative NF1 protein expression (χ2=20.732, *P*<0.001; Figure 2A). Furthermore, the log-rank test demonstrated that GC patients with low or negative expression of NF1 protein had a poorer prognosis than those with high NF1 expression (χ2=10.56, *P*=0.001).

### Expression level of NF1 mRNA in human gastric cancer tissues

Next, we performed real time quantitative PCR (qRT-PCR) to compare NF1 mRNA levels in gastric cancer and matched adjacent normal tissues. NF1 mRNA could be detected in both or one of the cancerous and matched adjacent normal tissues, or alternatively, none of the cancerous and adjacent normal tissues. The inter-assay coefficient of variation was <15% (in log scale) for this assay in our laboratory. Therefore, a 10-fold (one log) difference was not caused by inter-assay variations and should be considered an authentic difference in NF1 expression levels. All cancer tissues were grouped into three groups: NF1_high_, NF1_low_ and NF1_negative_ expression group, the cancer tissues were designated as negative expression when NF1 mRNA was undetectable in cancer samples, the cancer tissues were designed as low expression when NF1 mRNA was detectable but the level was 10-fold lower than that in the adjacent normal tissues, and the cancer tissues were designed as high expression when NF1 mRNA was detectable and the level was either higher than, equal to, or lower (but less than 10-fold difference) than that in the adjacent normal tissues.

**Figure 3:**
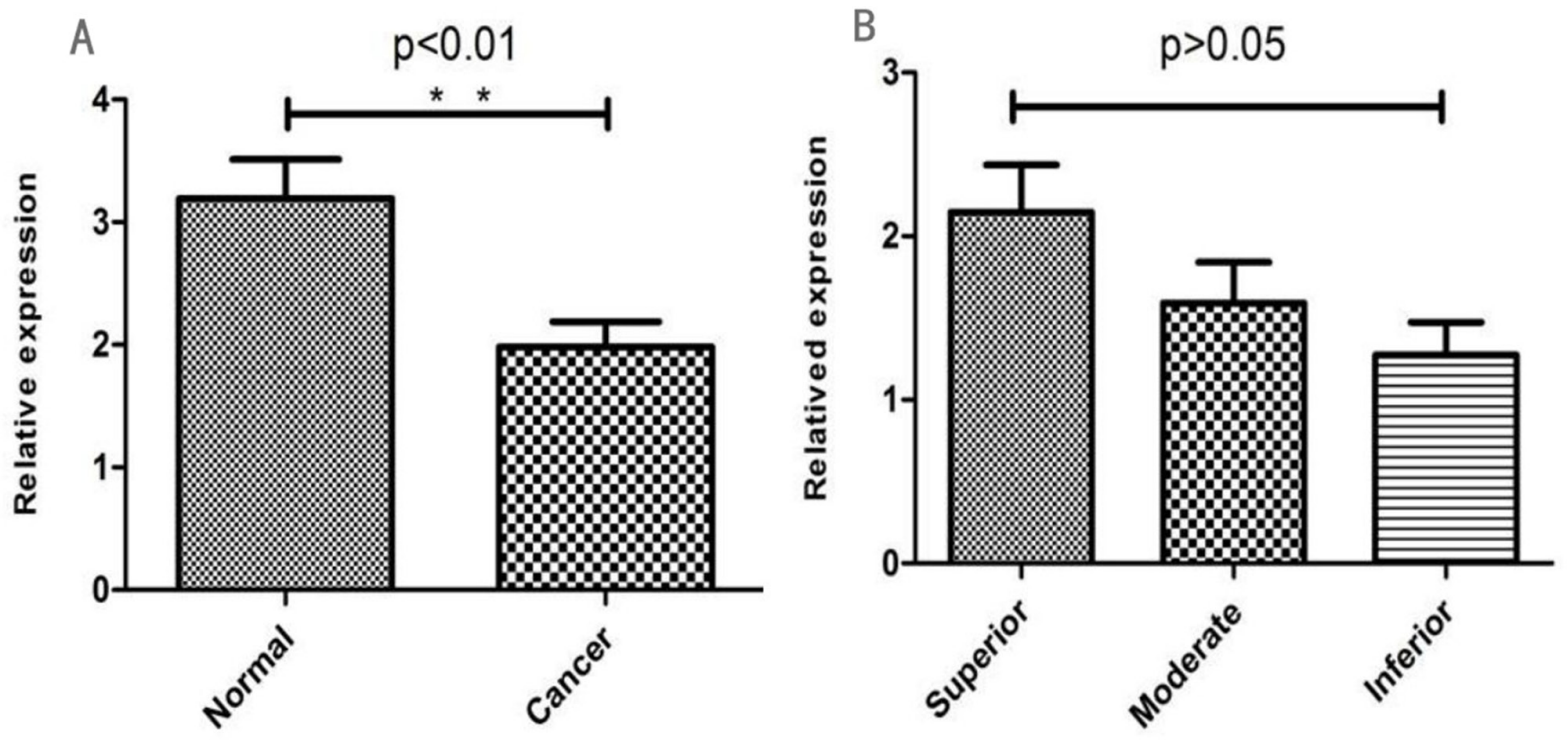
**(A)** Relative NF1 mRNA levels in normal and GC tissues (*P < 0.05; **P < 0.01). **(B)** Relative NF1 mRNA levels in different GC differentiation stages (*P < 0.05; **P < 0.01).

The qRT-PCR results showed that 96.3% (154/160) normal and 72.5% (116/160) gastric cancer tissues demonstrated NF1 mRNA expression (Table 3; Figure 3A). Our data showed that NF1 mRNA expression was significantly different between the gastric cancer and normal tissues (χ2=34.23, *P*<0.001). However, NF1 mRNA expression was similar among different differentiation groups (χ^2^=3.707, *P*>0.05; Figure 3B). These data further confirmed the immunohistochemical analysis, demonstrating that NF1 was highly expressed in normal tissues compared to gastric cancer tissues.

**Table 3:** The NF1 mRNA expression in normal and gastric cancer tissues

Tissue type	Total (n)	NF1 positive	% NF1 positives	*P value*
normal	160	154	96.3	<0.001
cancer	160	116	72.5	

### Relation between NF1 mRNA expression and clinicopathological features

When we compared NF1 mRNA expression with different clinicopathological features, we found no significant differences in relation to gender (*P*=0.141), age (*P*=0.800), tumor size (*P*=0.980), lymph-node metastasis (*P*=0.217), differentiation (*P*=0.476) and distant metastasis (*P*=0.416). However, NF1 mRNA levels correlated with T stage (χ^2^=16.04, *P*=0.014) and TNM stages (χ^2^=22.47, *P*<0.001; Table 4).

**Table 4:** Correlation between NF1 mRNA expression and clinicopathological characteristics of GC patients

Characteristics	n (%)	NF1 mRNA expression (case)			*P value*
		High (n=42)	Low (n=74)	Negative (n=44)	
Age (years)					
<60	64(40)	15	31	18	0.800
≥60	96(60)	27	43	26	
Gender					
Male	104(65)	25	54	25	0.141
Female	56(35)	17	20	19	
Tumor size (cm)					
<5	91(57)	23	41	27	0.980
≥5	69(43)	19	33	17	
Distant metastasis					
Yes	16(10)	2	9	5	0.416
No	144(90)	40	65	39	
T stage					
T1	16(10)	10	5	1	0.011
T2	21(13)	6	11	4	
T3	92(58)	22	43	27	
T4	31(19)	4	15	12	
N stage					
N0	48(30)	16	22	10	0.217
N1	63(40)	14	30	19	
N2	31(19)	9	15	7	
N3	18(11)	3	7	8	
Differentiation					
Superior	6(4)	2	1	3	0.476
Moderate	45(28)	14	21	10	
Inferior	109(68)	26	52	31	
TNM stage					
I	25(16)	15	8	2	<0.001
II	67(42)	17	27	23	
III	52(32)	8	30	14	
IV	16(10)	2	9	5	

### Relationship between NF1 mRNA expression and the prognosis of these gastric cancer patients

After a five-year follow-up, we found that patients with NF1 mRNA expression had better prognosis than those with negative NF1 mRNA expression (χ2=9.475, *P*=0.002). Furthermore, Kaplan–Meier analysis showed that patients with high NF1 mRNA expression had better overall survival than patients with low or negative NF1 mRNA expression (χ2=19.599, *P*<0.001; Figure 2B). These results showed that high NF1 mRNA expression indicated better prognosis for GC patients.

### NF1 expression was an independent variable closely related to patients’ survival

Further univariate and multivariate cox regression analysis showed that NF1 expression (HR, 1.57, 95%CI: 1.216-2.025; *P*=0.001) was an independent risk factor of survival of GC patients. Meanwhile, T stage (HR, 1.56, 95%CI: 1.203-2.023; *P*=0.001) was also found to be significantly associated with GC patients’ survival (Table 5).

**Table 5:** Univariate and multivariate cox analyses of survival in patients with GC

Tumor features	B	S.E.	Wald-χ2	*P*	RR (95%CI)
Univariate					
Gender	0.441	0.221	3.975	0.046	1.554(1.008-2.398)
Age	0.306	0.210	2.130	0.144	1.358(0.900-2.048)
Tumor size	0.406	0.191	4.507	0.034	1.501(1.032-2.184)
Differentiation	0.615	0.208	8.779	0.003	1.850(1.231-2.779)
T stage	0.506	0.127	15.878	<0.001	1.658(1.293-2.127)
N stage	0.282	0.097	8.510	0.006	1.326(1.097-1.603)
Distant metastasis	0.703	0.499	1.983	0.159	2.019(0.759-5.371)
TNM stage	0.394	0.107	13.452	0.001	1.483(1.201-1.831)
NF1 expression	0.531	0.128	17.115	<0.001	1.701(1.322-2.187)
Multivariate					
T stage	0.445	0.133	11.235	0.001	1.560(1.203-2.023)
NF1 expression	0.451	0.130	11.999	0.001	1.570(1.216-2.025)

## DISCUSSION

The *NF1* gene is one of the largest genes in the human genome, located at chromosome 17q11.2 and having 60 exons spanning over 350kb of genomic DNA [18, 19]. Recent studies have shown that *NF1* is a tumor suppressor that plays a critical role in many cancers [20, 21]. Mutations in *NF1* also lead to abnormalities in the cardiovascular, musculoskeletal and nervous systems [22]. Moreover, Iyengar *et al.* demonstrated that differential expression of the *NF1* isoforms was associated with cellular differentiation in ovarian epithelial cancer [23]. To our knowledge, the relationship between *NF1* and GC is unknown. Therefore, we systematically evaluated the expression of *NF1* in normal and cancer GC tissues and investigated its prognostic relevance in GC.

In the present study, immunohistochemical staining and qRT-PCR analysis demonstrated that NF1 protein and mRNA levels were significantly reduced in GC cancer tissues compared to normal tissues. Immunohistological staining showed that 94.4% normal tissue samples positively stained for NF1 protein compared to 58.1% in gastric cancer tissue samples. Further, low or negative NF1 protein expression was associated with T stage (*P*<0.05) and tumor node metastasis (*P*<0.001). Moreover, Kaplan-Meier survival analyses showed that patients with NF1 protein expression had better prognosis than those with low or negative NF1 protein expression (*P*<0.001). The qRT-PCR data corroborated these findings, and the univariate and multivariate cox regression analysis also demonstrated that NF1 expression was an independent risk factor of survival of GC patients, thereby suggesting that *NF1* was a potential prognostic marker for GC.

Many studies have highlighted the role of *NF1* in cancers. Yoon *et al.* reported that *NF1* gene product neurofibromin negatively regulated Ras and mammalian target of rapamycin (mTOR) signaling and prompted clinical trials to evaluate the ability of Ras and mTOR pathway inhibitors to arrest NF1-associated tumor growth [24]. Verhaak *et al.* demonstrated strong association between somatic *NF1* mutations and the mesenchymal subtype [23]. Elza *et al.* demonstrated that low NF1 levels were associated with primary and acquired resistance of lung adenocarcinomas to EGFR TKIs [25]. Another study showed that NF1 was an active regulator of GTP-Ras accumulation [26]. Also, loss of *NF1*gene lead to epithelial-mesenchymal transition (EMT), thereby implicating NF1 in tumorigenesis and cancer metastasis [27]. However, NF1 function in GC is unknown and needs to be investigated further.

Although our study demonstrated prognostic value of NF1 in GC patients, there are some limitations in our study. First, the gastric cancer sample size in our study is relatively small that may have introduced inherent bias. Hence, future studies with large-scale samples are necessary to confirm our findings. Second, we did not collect the disease free survival (DFS) data in our study, which could be influenced by the postoperative treatment and follow-up examinations. Third, we investigated NF1 gene expression in GC tissues and its association with patient prognosis. However, we did not investigate the mechanisms downstream of NF1. These mechanisms need to be deciphered in future investigations.

In conclusion, our study demonstrated that low or negative NF1 expression in human GC tissues was associated with higher TNM stage and poor five-year overall survival compared to those with high NF1 levels. These data indicated that NF1 was a potential prognostic indicator for the survival of GC patients.

## MATERIALS AND METHODS

### Patients and samples

The present study included primary gastric cancer specimens from 160 patients (104 males and 56 females), who underwent surgery at the Affiliated Hospital of Shandong Academy of Medical Sciences (Shandong, P.R. China) between August 2008 and June 2011. All patients in the present study were confirmed by pathological examination after surgery. The patients did not receive chemotherapy or radiotherapy before surgery. The clinical and pathological data of all patients was obtained by reviewing medical records and pathology reports. These included gender, age, tumor size, tumor differentiation, T stage, lymph node metastasis, distant metastasis, and tumor-node-metastasis (TNM) stage. Clinicopathological classification and staging was determined according to the criteria recommended by the American Joint Committee on Cancer (AJCC) [28]. The age of the participating patients ranged from 34 to 79 yrs (mean: 54 yrs). Normal tissue was also obtained from all patients and was at least 5cm away from the cancer tumors. Further, we obtained survival data from telephone or outpatient follow-ups. The last follow-up data was in May 2016. The median follow-up time was 45 months (range: 1–60 months). This study was reviewed and approved by the Ethics Committee of The Affiliated Hospital of Shandong Academy of Medical Sciences. We obtained written informed consent from all participants.

### Immunohistochemical staining

The normal and cancer tissue samples from 160 patients were paraffin embedded and cut into 4μm thick slices for immunohistochemical staining. Immunohistochemical staining was performed using the streptavidin-peroxidase two-stage method. Briefly, the tissue sections were dewaxed, hydrated and subjected to antigen retrieval with EDTA. Next, the samples were incubated with the rabbit anti-NF1 antibody (1:200; CUSABIO BIOTECH CO. Ltd) at 4°C overnight. This was followed by incubation with horseradish peroxidase-conjugated anti IgG antibody (1:1000; Santa Cruz, USA) for 45 min and three washes with PBS for 5 min each. Then, the samples were developed with diaminobenzidine solution for 3 min, washed briefly in running water, counter-stained with hematoxylin, dehydrated through a graded series of alcohol to xylene and sealed piece with a neutral gum. Negative control tissues were stained similarly except that the primary antibody was replaced with PBS.

### RNA extraction and real-time quantitative PCR (qRT-PCR)

Total RNA was extracted from cancer and non-cancerous tissues using Trizol (Invitrogen, USA) according to the instructions of the Reverse Transcription System kit (Vazyme Biotech, China). Reverse transcription was performed according to kit instructions followed by quantitative PCR using the Roche 480 II Real-Time PCR machine, and the real time PCR conditions and reaction mixture composition were showed in Tables 6 and 7. All the gene expression levels were analyzed and calculated by using the 2-ΔΔCt method [29]. NF1 and β-Actin primers were purchased from Sangon Biotech Company. The primer sequences for NF1 were: forward, 5′-ACACATGCAAAATGGGAACA-3′ and reverse, 5′-TGGGACATTCGCCTCTTAAC-3′. The primer sequences for β-Actin were: forward, 5′-AGCGAGCATCCCCCAAAGTT-3′ and reverse, 5′-GGGCACGAAGGCTCATCATT-3′.

**Table 6:** Real time PCR conditions

Program	Temperature	Time	Reaction times
Pre denaturation	95°C	10min	1
Denaturation	95°C	10s	
annealing	55°C	20s	40
extend	72°C	15s	
Insulation	72°C	3min	1

**Table 7:** Real time PCR reaction mixture composition

Reagents	Usage amount
SYBR® Premix Ex Taq II(Tli RNaseH Plus) (2×)	10 μl
PCR Forward Primer (10 μM)	0.8 μl
PCR Reverse Primer (10 μM)	0.8 μl
ROX Reference Dye or Dye II (50×)	0.4 μl
RT reaction solution (cDNA solution)	2 μl
dH2O (Sterilized distilled water)	6 μl
Total	20 μl*4

### Evaluation of NF1 immunohistochemical staining

Immunohistochemical stained tissue sections were observed and photographed with a light microscope (Carl Zeiss, Germany). NF1 was detected mainly in the cytoplasm of the normal and tumor cells. The NF1 immunohistochemical stained tissue sections were reviewed and assessed independently by three pathologists in a blinded manner and a consensus was reached for each score. Scoring was based on the percentage of stained tumor cells (1=< 10%; 2=11%-50%; 3=> 51%) in a given tumor sample and the intensity of cytoplasmic staining that was graded as negative (score 0), weak (score 1), moderate (score 2), or strong (score 3). The final NF1 staining scores were calculated as percentage × staining intensity. Hence, final scores were, score 0=value 0; score 1=value 1-3; score 2=value 4-6; score 3=value 7-9. Then, the tissue sections were divided into two groups, low NF1 (score 0 or 1) and high NF1 (score 2 or 3) groups.

### Statistical analysis

All data were statistically analyzed by SPSS version 17.0 software (SPSS Inc, Chicago, IL, USA). The Pearson’s Chi-squared (χ^2^) test was used to evaluate the difference between levels of NF1 staining in specimens and clinicopathological characteristics. The Kaplan-Meier survival analysis method was used to assess the association between NF1 expression and overall survival. The log-rank test was used to evaluate the differences between survival curve of patients with high or low NF1 expression. To determine prognostic factors, multivariate regression analysis was performed using the Cox proportional hazards model for variables with *P*<0.05 in the univariate Cox analyses. All *P* values were two-sided and a *P* value less than 0.05 were considered statistically significant.
